# Identification of disease-causing variants by comprehensive genetic testing with exome sequencing in adults with suspicion of hereditary FSGS

**DOI:** 10.1038/s41431-020-00719-3

**Published:** 2020-09-04

**Authors:** Matthias Christoph Braunisch, Korbinian Maria Riedhammer, Pierre-Maurice Herr, Sarah Draut, Roman Günthner, Matias Wagner, Marc Weidenbusch, Adrian Lungu, Bader Alhaddad, Lutz Renders, Tim M. Strom, Uwe Heemann, Thomas Meitinger, Christoph Schmaderer, Julia Hoefele

**Affiliations:** 1 Department of Nephrology, Klinikum rechts der Isar, School of Medicine, Technical University of Munich, Munich, Germany; 2 Institute of Human Genetics, Klinikum rechts der Isar, School of Medicine, Technical University of Munich, Munich, Germany; 3 Institute of Human Genetics, Helmholtz Zentrum München, Neuherberg, Germany; 4 Institute of Neurogenomics, Helmholtz Zentrum München, Neuherberg, Germany; 5 Nephrologisches Zentrum, Medizinische Klinik und Poliklinik IV, Klinikum der Universität München, Ludwig-Maximilians University, Munich, Germany; 6 Pediatric Nephrology Department, Fundeni Clinical Institute, Bucharest, Romania

**Keywords:** Focal segmental glomerulosclerosis, Genetics research, End-stage renal disease, Paediatric kidney disease

## Abstract

In about 30% of infantile, juvenile, or adolescent patients with steroid-resistant nephrotic syndrome (SRNS), a monogenic cause can be identified. The histological finding in SRNS is often focal segmental glomerulosclerosis (FSGS). Genetic data on adult patients are scarce with low diagnostic yields. Exome sequencing (ES) was performed in patients with adult disease onset and a high likelihood for hereditary FSGS. A high likelihood was defined if at least one of the following criteria was present: absence of a secondary cause, ≤25 years of age at initial manifestation, kidney biopsy with suspicion of a hereditary cause, extrarenal manifestations, and/or positive familial history/reported consanguinity. Patients were excluded if age at disease onset was <18 years. In 7/24 index patients with adult disease onset, a disease-causing variant could be identified by ES leading to a diagnostic yield of 29%. Eight different variants were identified in six known genes associated with monogenic kidney diseases. Six of these variants had been described before as disease-causing. In patients with a disease-causing variant, the median age at disease onset and end-stage renal disease was 26 and 38 years, respectively. The overall median time to a definite genetic diagnosis was 9 years. In 29% of patients with adult disease onset and suspected hereditary FSGS, a monogenic cause could be identified. The long delay up to the definite genetic diagnosis highlights the importance of obtaining an early genetic diagnosis to allow for personalized treatment options including weaning of immunosuppressive treatment, avoidance of repeated renal biopsy, and provision of accurate genetic counseling.

## Introduction

The advances made possible by next-generation sequencing (NGS) techniques have shown that in about 30% of infantile, juvenile, or adolescent patients with steroid-resistant nephrotic syndrome (SRNS), a monogenic cause can be identified [1]. The histological correlate in patients with SRNS is often focal segmental glomerulosclerosis (FSGS) or minimal change disease (MCD) in early stages [2]. So far, more than 50 genes have been associated with hereditary FSGS/SRNS [3–7]. The clinical manifestation of hereditary FSGS is extremely variable with differences in expressivity, apparent by different ages of disease onset, progression to end-stage renal disease (ESRD) or the presence of nephrotic syndrome (NS). NS is defined by significant proteinuria (>40 mg/m^2^ per hour). Patients with NS typically present with hypoalbuminemia causing severe edema. Progression of chronic kidney disease gradually leads to ESRD requiring dialysis or transplantation. Most importantly, this has a strong impact on patient survival. In contrast, in adult patients with FSGS, SRNS, or NS, hereditary causes are widely neglected differential diagnoses.

Genetic studies have assigned a pivotal role to the podocyte in terms of pathophysiology of hereditary FSGS/SRNS. Multiple genes encoding slit membrane components, actin binding proteins, proteins important for coenzyme Q_10_ biosynthesis, as well as nuclear transcription factors have been associated with monogenic-caused podocyte dysfunction [5]. These genes are highly expressed in the podocyte leading to their degeneration with consecutive symptoms in the presence of disease-causing variants [likely pathogenic or pathogenic variants as per the American College of Human Genetics (ACMG) with a fitting genotype]. Furthermore, variants in the *COL4A3-5* genes, encoding elements of the glomerular basement membrane and associated with Alport syndrome, can mimic the histological and/or clinical picture of FSGS/SRNS [8, 9]. Most literature is limited due to the use of panel diagnostics or targeted single-gene sequencing. The heterogeneous presentation in hereditary FSGS/SRNS favors a hypothesis-free approach like exome or genome sequencing (ES or GS) to adequately address its genetically heterogenous nature.

It is assumed that in at least 10% of adults who require renal replacement therapy a hereditary cause can be identified. This would represent one of the most common causes of ESRD next to diabetes, hypertension, glomerulonephritis, and pyelonephritis [10]. The likelihood for identifying a disease-causing variant resulting in FSGS/SRNS is inversely related to age of onset due to usually early manifestation in monogenic cases [1, 5, 11, 12]. The diagnostic yield in patients above 21 years at study entry with glomerulopathies ranges from 7% in unselected cohorts [13] to 12–14% in selected cohorts [14, 15]. Overall, genetic and phenotypic data on patients with FSGS or SRNS disease onset above 18 years are scarce. Only few monogenic causes have been identified in adult patients, with a mean FSGS disease onset ranging from 26 years in patients with disease-causing variants in classical FSGS genes to 36 years in patients with disease-causing variants in *COL4A3-5* [16].

Using ES in a cohort of 24 adult patients with suspected hereditary FSGS/SRNS, we aimed to identify disease-causing variants.

## Material and methods

### Study population

The study was approved by the local Ethics Committee of the Technical University of Munich and performed according to standards of the 2013 Helsinki Declaration. Written informed consent was obtained from patients or their legal guardians.

For inclusion, a selection process was applied for adult disease onset patients with assumed hereditary FSGS or MCD to increase the likelihood for the identification of a monogenic cause. At least one of the following criteria had to be met to perform ES: absence of a secondary cause for FSGS, ≤25 years of age at initial manifestation, kidney biopsy with suspicion of a hereditary cause (i.e., ultrastructural changes of the glomerular basement membrane in electron microscopy [17]), extrarenal manifestations (syndromal disease, for example intellectual disability, eye or skeletal abnormalities), and/or positive familial history/reported consanguinity. Exclusion criteria were missing written informed consent, age below 18 years at initial manifestation of kidney disease, and absence of the above-mentioned inclusion criteria.

**Fig. 1:**
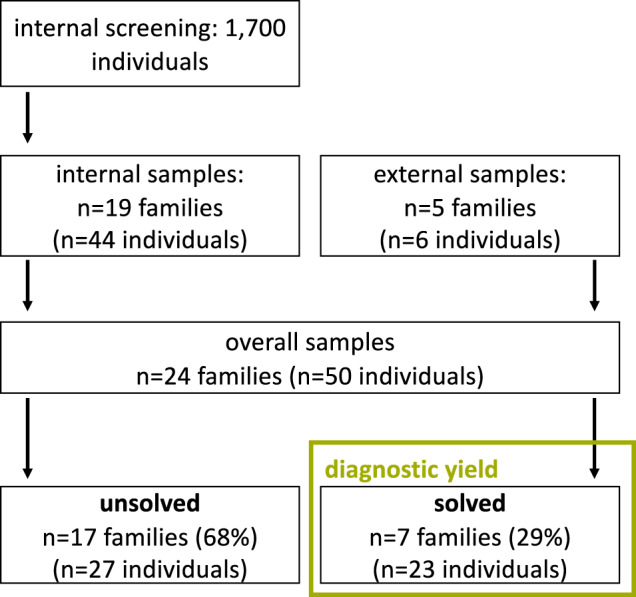
Flow chart, cohort overview. A total of 1700 in-house individuals were screened for suspected hereditary FSGS. Decision to include patients was based on prioritization criteria, which led to the inclusion of 19 index patients. In addition, five samples were sent in for molecular analysis. A total of 29% of the cases could be solved. A total of 68% of the patients did not show any clear disease-causing variant.

To identify patients with a high likelihood for a hereditary cause, we performed a screening of 1700 adult kidney disease patients treated at our university hospital between January 2000 and February 2018. In addition, we received external samples meeting the inclusion criteria (Fig. 1). Origin was classified into European (non-Finnish) and other origins.

### Clinical case information

Clinical and phenotype information was retrieved from clinical reports and medical history. Age of disease onset was defined as the earliest report of a kidney disease in the medical history (e.g., first onset of proteinuria, edema, hospitalization due to a kidney disease, etc.). Age of onset of ESRD was determined as the beginning of renal replacement therapy (hemodialysis or peritoneal dialysis) or preemptive kidney transplantation. A standardized questionnaire was used for the assessment of clinical information. Stage of chronic kidney disease was assessed at the latest available date according to KDIGO guidelines [18].

### Genetics

DNA was extracted from peripheral blood using the Gentra Puregene Blood Kit (Qiagen, Hilden, Germany) according to the manufacturer’s instructions. ES was performed using a Sure Select Human All Exon 60 Mb V6 Kit (Agilent) and a HiSeq4000 (Illumina) or a Sure Select Human All Exon 60 Mb V5 Kit (Agilent) and a HiSeq2500 (Illumina) as previously described [19]. Mitochondrial DNA was derived from off-target exome reads as previously described [20]. Reads were aligned to the human reference genome (UCSC Genome Browser build hg19) using Burrows-Wheeler Aligner (v.0.7.5a). Detection of single-nucleotide variants and small insertions and deletions (indels) was performed with SAMtools (version 0.1.19). Exome depth was used for the detection of copy-number variations [21]. Confirmation of the identified variants and segregation analysis was carried out by Sanger sequencing. For the analysis of *de novo*, autosomal dominant and mitochondrial variants, only variants with a minor allele frequency of less than 0.1% in the in-house database of the Helmholtz center Munich consisting of over 16,000 exomes were considered. For the analysis of autosomal recessive and X-linked variants [homozygous, hemizygous, or (putatively) compound heterozygous], only variants with a minor allele frequency of less than 1% were considered. For the *NPHS2* p.(Arg229Gln) allele a MAF > 1% was accepted due to the known association with hereditary nephropathy when found in trans with a (likely) pathogenic variant [22, 23]. Furthermore, variants were compared to publicly available databases such as the Genome Aggregation Database (gnomAD). Only variants rated as “likely pathogenic” or “pathogenic” according ACMG classification and recent amendments and with a genotype in agreement with the mode of inheritance led to the designation “solved case” [24, 25]. To confirm if a variant was already published, a data search for the respective variant was performed in PubMed, ClinVar, and the Human Gene Mutation Database.

In addition, in one case (F332) with positive familial history and suspected autosomal dominant inheritance, *MUC1* analysis was performed as previously described by Southern blot after unremarkable ES [26, 27].

Identified variants were submitted to the Leiden Open Variation Database (LOVD; https://databases.lovd.nl/shared) and to ClinVar (https://www.ncbi.nlm.nih.gov/clinvar/).

### Statistics

Categorical data are presented as absolute and relative frequencies. Continuous variables are expressed as median and interquartile range (IQR, 25th–75th percentile). For the calculation of the diagnostic yield only pathogenic and likely pathogenic variants with a fitting genotype (disease-causing variants) according to the definition of the ACMG standards and guidelines (i.e., classified as “solved cases”) were used. To test for group differences *t*-test was used and presented as mean ± standard deviation. To examine disease and ESRD onset as well as duration until the definite genetic diagnosis only “solved cases” were considered. All statistical tests were two-sided and *p* values < 0.05 were considered significant. Statistical analysis was performed using SPSS version 23.0 (SPSS Inc., Chicago, IL, USA).

## Results

A total of 1700 in-house patients were screened and 19 index patients were included into the study. These index patients came from 19 unrelated families comprising a total of 44 individuals. Furthermore, five index patients from unrelated families comprising six individuals were submitted from external cooperation partners (Fig. 1).

Overall, 37 (74%) of the total 50 individuals were affected. Overall, 26 (70%) of these 37 affected individuals were male and 11 (30%) were female. Reported consanguinity was present in 2 (8%) families. Overall, 20 (83%) families were of European (non-Finnish) origin.

Of the overall 24 index patients, trio ES was done in three index cases (13%), duo ES in two (8%), and singleton ES in 19 (79%). In seven index patients, a disease-causing variant could be identified, corresponding to a total diagnostic yield of 29%. Overall, 5/7 index patients with a disease-causing variant were internal samples (diagnostic yield of the internal samples: 26%). Overall, 17 (71%) index patients could not be solved genetically.

### Genetic variants

Overall, eight different variants were present in six known disease-causing genes (Table 1). Six of these variants have been described before as disease-causing. Overall, 2 (22%) variants, so far not described before, could be classified as disease-causing (likely pathogenic and pathogenic) according to ACMG guidelines (Table 1): the variant in *COL4A3*, c.2126-1G>C, p.(?) was present in a compound-heterozygous state with the variant c.4421T>C, p.(Leu1474Pro) and absent in gnomAD. The heterozygous variant in *COL4A5*, c.2359G>A, p.(Gly787Arg), was absent in our in-house database as well as in gnomAD. All identified variants reported in this study were at least conserved until M. musculus. In unsolved cases, no disease-causing variants could be detected in known disease-associated genes and no variants in candidate genes could be prioritized.

**Table 1:** Overview of identified variants.

Family (origin)	Gene (NM_number)	Chromosomal position (hg19)	Nucleotide change, amino acid change, and corresponding ClinVar and/or LOVD ID	Inheritance	gnomAD^a^ MAF	ACMG rating^b^	Biopsy (initial clinical presentation)	Affected (gender)	Positive familial history	Consanguinity	Individual ages (years)		CKD-stage (age in years)	Present inclusion criteria^c^	Previously published
											Disease onset	ESRD			
F9 (GE)	*COL4A3* (NM_000091.4)	NC_000002.11: g.[228144508G>C]; [228172594T>C]	c.[2126-1G>C];[4421T>C], p.[?];[Leu1474Pro] LOVD ID: 0000665278; ClinVar ID: SCV001149720.1	AR	Not listed; 2.66e−3	PVS1/PM2, PS4 (moderate, in trans with pathogenic variant)/ PM1/PM3/PP3	FSGS (NS)	1 (m)	No	No	26	–	G3aA3 (30)	1	No; [42, 43]
F274 (GE)	*COL4A5* (NM_000495.3)	NC_000023.10: g.[107850086G>A]; [=]	c.[2359G>A];[=], p.[Gly787Arg];[=] ClinVar ID: SCV001150068.1	XL	Not listed	PS2/PM1 (strong)/PM2/PM5/PP3	Focal segmental and global GS (hypertension)	1 (f)	No	No	32.5	–	G3aA3 (40)	1, 3	No
F26 (TU)	*COQ8B* (NM_024876.4)	NC_000019.9: g.[41198128C>A]; [41198128C>A]	c.1447G>T(;)(1447G>T), p.Glu483*(;)(Glu483*) ClinVar ID: SCV001149996.1	AR	5.16e−5 (no homozygotes present)	PVS1/PS4 (moderate)/ PM2	FSGS (proteinuria)	2 (m, f)	Yes	Yes	24, 32	24, –	G5-D (24), G2A3 (32)	1, 5, 6	[38]
F103 (GE)	*INF2* (NM_022489.3)	NC_000014.8: g.[105169540G>C]; [=]	c.[490G>C];[=], p.[Ala164Pro];[=] LOVD ID: 0000665276	AD	Not listed	PM1/PM2/PP1 (strong)/PP3/	Minimal change disease, mesangioprol. GN IgA type (proteinuria, hypertension)	10 (m, m, f, f, f, m, m, m, m, m)	Yes	No	18, 19, 24, 25, 18, 32, 32, 19, 31, 26	30, 39, –, –, 38, 38, –, –, –, –	G5-D (30), G5-D (39), G1A? (39), G2A? (35), G5-D (38), G5-D (38), n.k., G4A3 (38), G3bA3 (32), G2A3 (27)	1, 2, 5	[44]
F27 (GE)	*INF2* (NM_022489.3)	NC_000014.8: g.[105169653C>T]; [=]	c.[529C>T];[=], p.[Arg177Cys];[=] ClinVar ID: SCV001149810.1	AD	Not listed	PS4 (moderate)/PM1/PM2/PP3	IgA/Immune complex nephritis, FSGS^d^ (proteinuria)	1 (m)	No	No	18	–	G4A3 (37)	2	[45]
F332 (GE)	*MUC1* (NM_001204285.1)	NC_0000001.10: g.(155160963_155162030)insC	c.(103_564)insG;[=], p.[?];[=] LOVD ID: 0000673664	AD	Not listed	PS3/PS4 (moderate)/PM2	Focal global GS (creatinine increase)	1 (m)	Yes	No	32	41	G5-D (41)	1, 5	[26]
F520 (RO)	*WT1* (NM_024426.4)	NC_000011.9: g.[32413514G>A]; [=]	c.[1432+4C>T];[=], p.[?];[=] LOVD ID: 0000673826	AD	Not listed	PS3/PS4 (moderate)/PM2/PP3	FSGS (edema)	1 (m [f])^e^	No	No	18	18	G5-D (18)	1, 2, 4	[46]

*P* pathogenic, *PP* pathogenic supporting, *VS* very strong, *S* strong, *M* moderate, *A?* albuminuria unknown, *AR* autosomal recessive, *AD* autosomal dominant, *ClinVar ID* https://www.ncbi.nlm.nih.gov/clinvar/, *CKD V-D* chronic kidney disease stadium V-dialysis, *XL* X-linked, *ESRD* end-stage renal disease, *FSGS* focal segmental glomerulosclerosis, *GE* Germany, *LOVD ID* https://databases.lovd.nl/shared, *MAF* minor allele frequency, *NS* nephrotic syndrome, *n.k.* not known, – not applicable, *RO* Romania, *TU* Turkey.

^a^Genome Aggregation Database (https://gnomad.broadinstitute.org/).

^b^See [24, 25].

^c^Inclusion criteria: 1: absence of a secondary cause for FSGS; 2: age ≤ 25 years at initial manifestation; 3: kidney biopsy with suspicion of a hereditary cause; 4: extrarenal manifestation; 5: positive familial history; and 6: reported consanguinity.

^d^Initial biopsy IgA/immunocomplex nephritis, second biopsy 7 years later FSGS.

^e^Genetic sex is a male karyotype (46,XY), the phenotype is female with unfulfilled desire to have children.

### Age of disease and ESRD onset, and time to definite genetic diagnosis

The median age of disease onset and of ESRD of all 37 affected individuals was 26.0 [19.4–32.3] and 38.0 [28.4–39.6] years, respectively. In patients with a disease-causing variant, median age of disease onset and of ESRD was 25.0 [18.5–32.0] and 38.0 [24.0–39.0] years, respectively. Independent *t*-test showed that age of disease onset was not significantly different in solved versus unsolved cases (25.1 ± 5.9 vs 31.5 ± 14.3 years; *p* = 0.09). Similarly, age at ESRD did not differ significantly between solved and unsolved cases (32.6 ± 8.8 vs 44.9 ± 16.4 years; *p* = 0.15).

Overall, 15 (63%) out of 24 index patients had a disease onset ≥ 25 years. Of these 15 patients with disease onset ≥ 25 years, in 4 (27%) patients, a disease-causing variant was detected, and in 11 (73%) patients, no disease-causing variant could be identified.

Looking at the overall time to diagnosis in solved cases from the first manifestation of a renal disease to the definite genetic diagnosis the median time was 9.0 [2.9–19.0] years, with a maximum of 37 years and a minimum of 0 years (i.e., predictive molecular analysis in several family members of F103).

### Clinical presentation and biopsy result

**Fig. 2:**
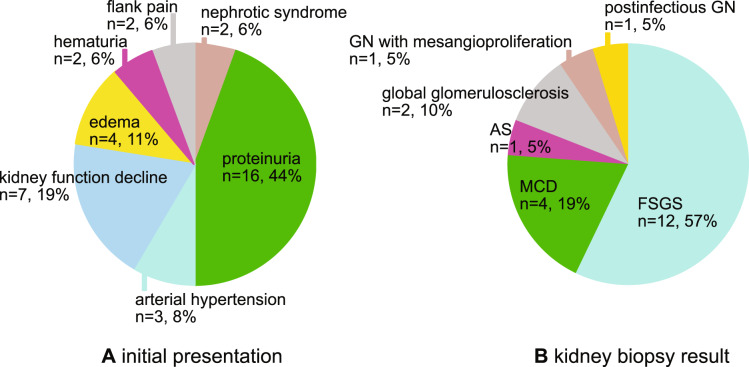
Pie chart, initial presentation, and kidney biopsy result of the affected patients. **a** The initial clinical presentation of the affected individuals. **b** The kidney biopsy results available from 21 affected individuals. AS Alport syndrome, FSGS focal segmental glomerulosclerosis, GN glomerulonephritis, MCD minimal change disease.

Overall, in all 37 affected individuals, the most frequent initial clinical presentation was proteinuria in 16 (44%) patients, followed by decline in kidney function in 7 (19%), edema in 4 (11%), and arterial hypertension in 3 (8%). Further causes were NS (*n* = 2; 6%), flank pain/nephrolithiasis (*n* = 2; 6%), or hematuria alone or combined with proteinuria (*n* = 2; 6%). In one affected sibling no data was available (Fig. 2a).

Concerning the cases in which a genetic diagnosis was made, 9 (64%) patients presented with proteinuria, and 2 (14%) patients with decline in kidney function, followed by NS (*n* = 1; 7%), arterial hypertension (*n* = 1; 7%), and edema (*n* = 1; 7%).

From the overall sample of all affected individuals, in 21 (57%) patients a kidney biopsy was available. Most frequent histological results showed FSGS in 12 (57%) patients or MCD in 4 (19%) patients. Other findings were global glomerulosclerosis (*n* = 2; 10%), lesions compatible with AS (*n* = 1; 5%), glomerulonephritis with mesangioproliferation (*n* = 1; 5%), and postinfectious glomerulonephritis (*n* = 1; 5%) (Fig. 2b).

## Discussion

We were able to identify a disease-causing variant by ES in 29% (7/24) of adult patients with clinically suspected hereditary FSGS. This diagnostic yield is comparable to studies performed in children or adolescent patients [1]; however, it is so far unparalleled in adult patients. Compared to cohorts with a similar age structure, our diagnostic yield of 29% in patients with disease onset ≥18 years is quite high [14–16]. This high diagnostic yield could be explained by several factors.

Adult patients were included based on specific criteria. This selection process allowed us to include patients with disease onset ≥ 18 years of age while maintaining a potential high likelihood for the identification of a monogenic cause. Compared to the overall number of in-house screened patients, 19 included in-house index patients represent a stringently selected cohort. In all selected patients, comprehensive genetic testing by ES was performed, therefore, enabling the identification of variants throughout the exome instead of focusing on a limited set of genes via panel diagnostics. For future investigations, we plan on creating a scoring system based on the above-mentioned criteria for the identification of patients with a high likelihood of a hereditary nephropathy in adult age of onset.

Interestingly, in a cohort (*n* = 135) of only nonsyndromic FSGS and/or SRNS adult cases without family history, a quite high diagnostic yield of 12% has been reported [15]. This highlights the importance of hereditary causes in sporadic cases because of *de novo* variants. Trio ES was only performed in 13% of our cohort.

In addition to the high diagnostic yield in our adult patient cohort, we could identify two novel (likely) pathogenic variants in known disease-causing genes.

In our cohort, genetic heterogeneity is illustrated by three cases with a different genetic diagnosis than initially clinically presumed (FSGS-mimics/-phenocopies) (three out of seven solved cases; 43%). Two out of these three cases (F9 and F274) had disease-causing variants in the *COL4A* genes that have been frequently associated with hereditary FSGS [8, 9, 28]. Furthermore, one case with FGGS had a pathogenic variant in *MUC1*. Two of these three cases should be highlighted: the index patient, in whom a heterozygous disease-causing variant in *COL4A5* was identified (F274), was female. She presented without the full picture of Alport syndrome but chronic renal failure with FSGS/FGGS on biopsy illustrating the wide range of phenotypes of X-linked Alport syndrome in females. Furthermore, we identified a cytosine insertion in the VNTR region of *MUC1* (on additional targeted testing), which has been associated with ADTKD-*MUC1*; however, our patient (F332) presented with a creatinine increase and FGGS in the kidney biopsy, which has been performed late in the disease course, i.e., 3 years before preemptive living kidney donation. Although, in this case, F332, kidney biopsy showed FGGS rather than FSGS, the family was still included in this study as in the progress of the disease a more diffuse and global pattern of sclerosis can occur [29, 30]. Furthermore, there was a high likelihood of a hereditary kidney disorder as several individuals in the family were affected (Table 1). These two cases show that (i) the unbiased approach of ES can help to unravel cases with an ambiguous phenotype (F274), and (ii) limitations of ES should be known and additional methodologic approaches employed as, for example, the cytosine insertion in the VNTR region of *MUC1* cannot be identified by ES (F332). The presence of FSGS-mimics/-phenocopies emphasizes that histologically diagnosed FSGS/FGGS can represent the final common track of several hereditary kidney disease entities. This is also underlined by a recently published case of a misclassification of ADTKD-*UMOD* as FSGS [31].

### Clinical implications

The long period of 9 years until the final genetic diagnosis in patients with disease onset ≥ 18 years of age could be made highlights the importance of routinely including the possibility of a hereditary cause in the differential diagnosis of FSGS also in adult patients. Patients with a disease-causing variant reached ESRD earlier than unsolved cases; however, the difference was not significant. Larger studies need to evaluate if the presence of a disease-causing variant leads to earlier onset of ESRD. Furthermore, the long delay until a genetic diagnosis was made could be explained by three factors: (i) the presence of FSGS/SRNS-mimics/-phenocopies which makes it difficult to classify the clinical picture; (ii) the reluctant application of molecular techniques (e.g., NGS), which are still not widely used in clinical routine, at least in adult nephrology, and (iii) the missing awareness for hereditary causes of chronic kidney disease in adult patients.

In general, the histological term “FSGS” applied for primary (idiopathic, immunological), secondary, and hereditary causes represents a very broad disease spectrum [29]. As seen in our study, selection of patients is challenging especially in adult disease onset patients due to possible discrepancies of the histological description and the molecular genetic diagnosis. In some biopsies (see Table 1), the histological picture did not fit the molecular genetic diagnosis or was different in consecutive biopsies (for example, F103 and F27). This phenotypic variability further highlights the precise definition of an underlying genetic cause by molecular testing in patients with a clinically presumed hereditary disease. In our cohort, in genetically diagnosed cases, only one patient initially presented with NS typical for FSGS. The most common initial presentation was non-nephrotic proteinuria (64%). Therefore, NS associated with disease-causing variants in FSGS genes could be primarily present in cases with early childhood onset [32]. However, it has to be noted that only three of the six identified causative variants were located in classic FSGS genes. And, interestingly, case F9 had the genetic diagnose of Alport syndrome but the unusual adult manifestation of NS.

In terms of treatment and management the number of hereditary causes detected in children with SRNS is much higher than in adult patients [33]. In SRNS, the long period of time between the first disease manifestation and the detection of the definite genetic diagnosis increases the risk of unnecessary immunosuppressive treatment. This immunosuppressive treatment is known to be effective in a subset of patients with SRNS but patients with a hereditary cause do not benefit from it [34]. Furthermore, in families with an index case and confirmed genetic diagnosis, the selection of a relative as a kidney donor should be evaluated carefully because in some hereditary causes, disease up to ESRD can occur later in life [35].

Furthermore, there is a small cohort of monogenic disorders that can be influenced by treatment. These include patients with pathogenic variants in the genes encoding enzymes of the coenzyme Q_10_ biosynthesis [36–38]. In our cohort, two affected individuals of one consanguineous family had a disease-causing variant in *COQ8B*. Several case reports suggest a beneficial effect of coenzyme Q_10_ supplementation [38, 39].

In general, an early genetic diagnosis could allow a personalized treatment approach with weaning of immunosuppressive treatment, avoidance of renal biopsy, and provision of accurate genetic counseling [40]. Furthermore, genetic diseases can have multisystemic complications that need to be taken into consideration by the clinician, which is illustrated by F520 where the presence of Frasier syndrome is associated with an increased risk for gonadoblastoma [41].

Several limitations have to be mentioned. The number of affected individuals is rather small, therefore, a statement on the age of disease onset, ESRD, initial presentation as well as the kidney biopsy results is limited and not generalizable. In addition, patients in this study were included mainly in the way of a retrospective case selection. Therefore, data on the course of the immunosuppressive treatment as well as on steroid-resistance vs steroid-sensitivity were not readily available. Due to a pragmatic and time-efficient approach for scoring the 1700 in-house screened patients, data about the distribution of primary versus secondary FSGS were not systematically assessed. Furthermore, precise data concerning family history or consanguinity were not documented in most of the 1700 in-house screened patients, potentially leading to the relatively low number of only 1.1% included patients. Therefore, independent cohorts are needed to evaluate if the inclusion criteria used in this study do increase the likelihood for the presence of monogenic disease cause when selecting patients for molecular genetic testing.

## Conclusion

We were able to identify a monogenic cause in more than one fourth of selected adult patients with suspected hereditary FSGS. This high diagnostic yield could only be achieved by including patients with a high likelihood for a hereditary disease cause based on specific selection criteria. The long time period until genetic diagnosis highlights the importance of comprehensive genetic testing, e.g., ES, to obtain an early genetic diagnosis that allows a personalized treatment approach with weaning of immunosuppressive treatment, avoidance of renal biopsy, and provision of accurate genetic counseling.

## Supplementary information

Supplemental Material
