# Supplementary material for: Identification of disease-causing variants by comprehensive genetic testing with exome sequencing in adults with suspicion of hereditary FSGS
Source: Eur J Hum Genet. 2020 Sep 4;29(2):262–70. doi: 10.1038/s41431-020-00719-3 (PMC7868362; doi:10.1038/s41431-020-00719-3)
Supplement: Supplementary file 1 — Supplemental Material [file 41431_2020_719_MOESM1_ESM.docx]

**Supplementary Material**

**Identification of Disease-Causing Variants by Comprehensive Genetic Testing with Exome Sequencing in Adults with Suspicion of Hereditary FSGS**

**Matthias Christoph Braunisch^1,2^, Korbinian Maria Riedhammer^1,2^, Pierre-Maurice Herr^2^, Sarah Draut^2^, Roman Günthner^1,2^, Matias Wagner^2,3,4^, Marc Weidenbusch^5^, Adrian Lungu^6^, Bader Alhaddad^2^, Lutz Renders^1^, Tim M Strom^2,3^, Uwe Heemann^1^, Thomas Meitinger^2,3^, Christoph Schmaderer^1^, and Julia Hoefele^2^**

Table of Contents

[Pedigree and segregation F9, *COL4A3* (NM_000091.4) 2](#_Toc39658781)

[Pedigree and segregation F26, *COQ8B* (NM_024876.4) 2](#_Toc39658782)

[Pedigree and segregation F27, *INF2* (NM_022489.3) 3](#_Toc39658783)

[Pedigree and segregation F103, *INF2* (NM_022489.3) 4](#_Toc39658784)

[Pedigree and segregation F274, *COL4A5* (NM_000495.3) 5](#_Toc39658785)

[Pedigree and segregation F332, *MUC1* (NM_001204285.1) 5](#_Toc39658786)

[Pedigree and segregation F520, *WT1* (NM_024426.4) 6](#_Toc39658787)

# Pedigree and segregation F9, *COL4A3* (NM_000091.4)

c.[2126-1G>C];[4421T>C], p.[?];[Leu1474Pro]


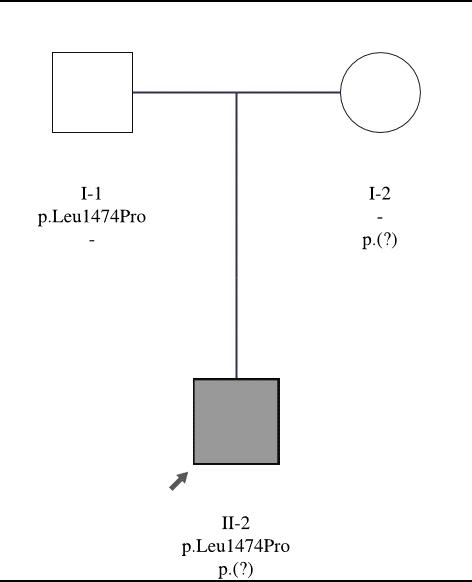


# Pedigree and segregation F26, *COQ8B* (NM_024876.4)

c.1447G>T(;)(1447G>T), p.Glu483*(;)(Glu483*)


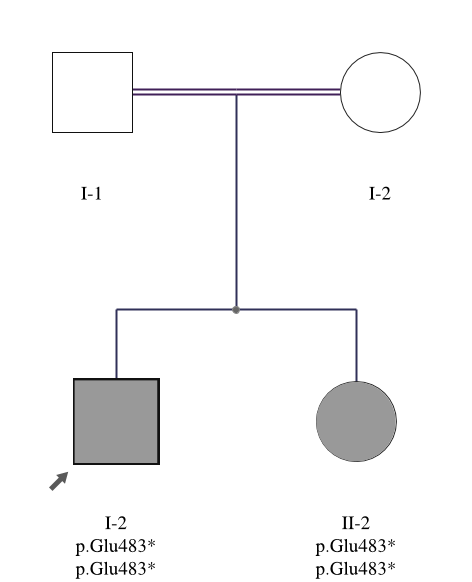


# Pedigree and segregation F27, *INF2* (NM_022489.3)

c.[529C>T];[=], p.[Arg177Cys];[=]


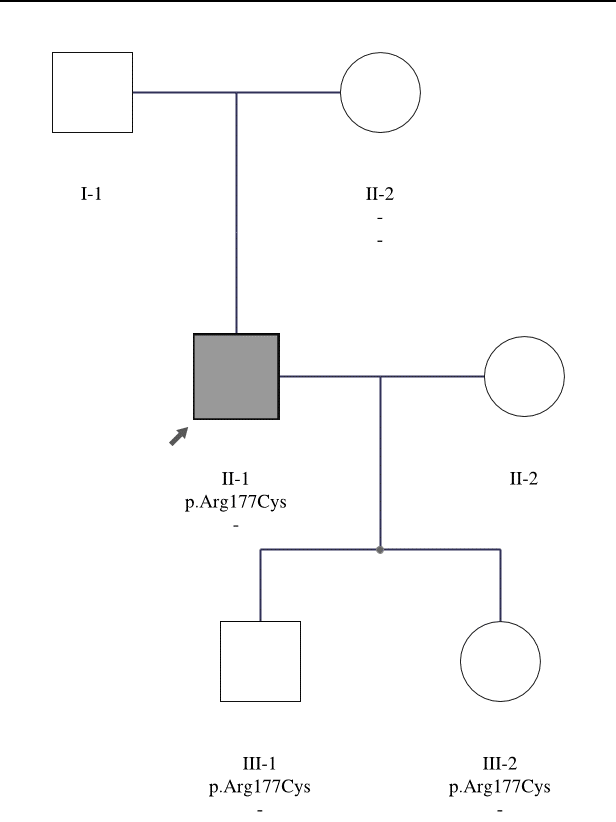


Note: Predictive testing was performed in the two asymptomatic children III-1 and III-2. III-1 is presently 9 years old and III-2 is presently 6 years old. Disease-causing variants are typically associated with a late-onset phenotype ^1^.

# Pedigree and segregation F103, *INF2* (NM_022489.3)

c.[490G>C];[=], p.[Ala164Pro];[=]


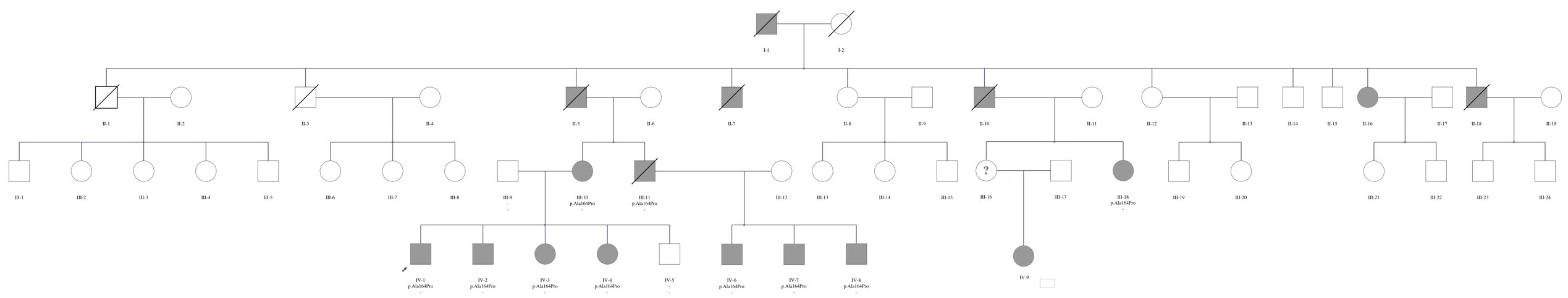


Note: No DNA and phenotype information were available from III-16. Also, no DNA was available from IV-9.

# Pedigree and segregation F274, *COL4A5* (NM_000495.3)

c.[2359G>A];[=], p.[Gly787Arg];[=]


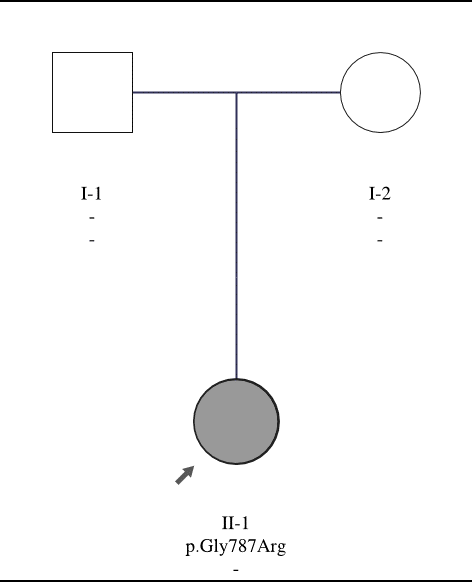


# Pedigree and segregation F332, *MUC1* (NM_001204285.1)

c.[103_564insG];[=], p.[?];[=]


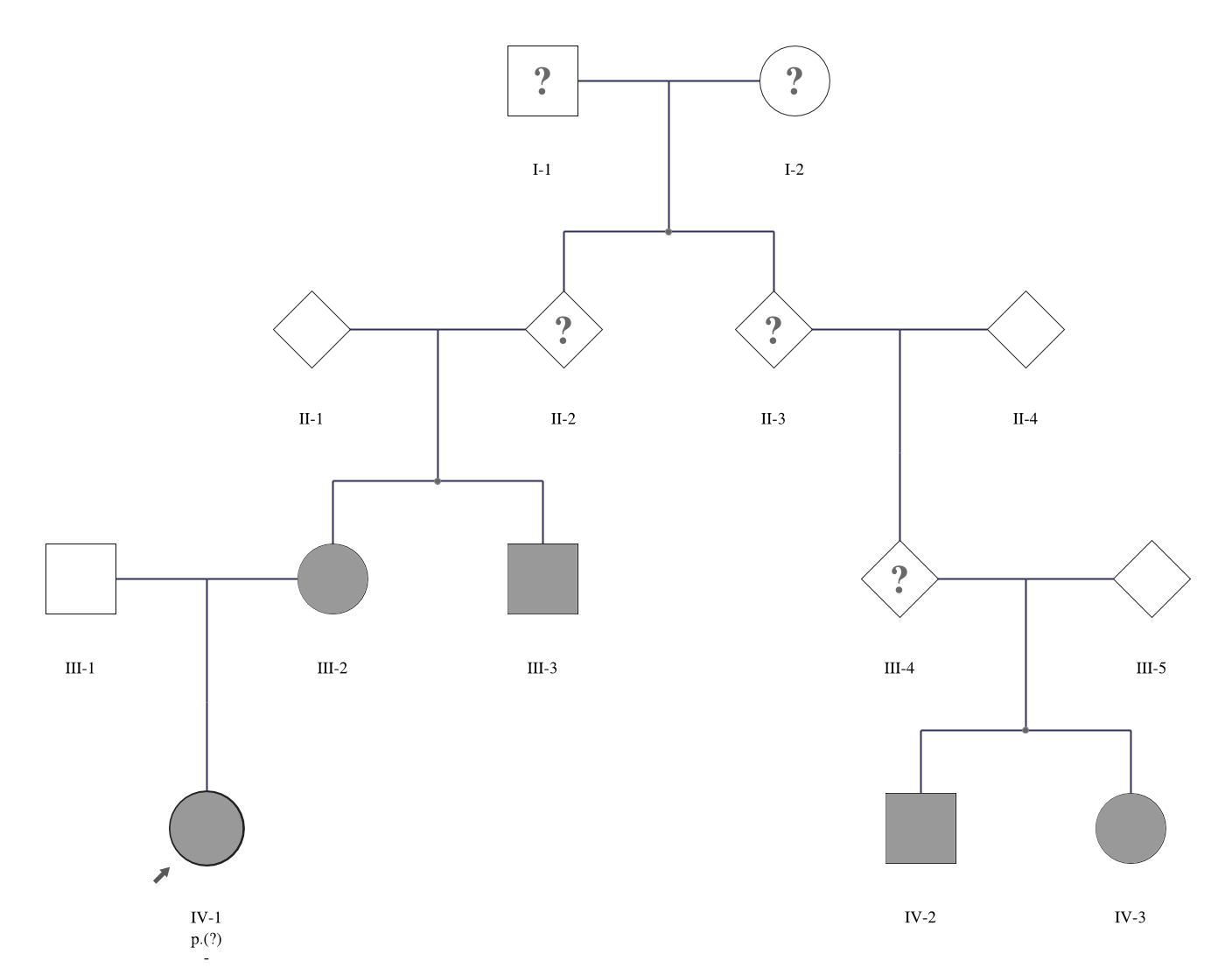


# Pedigree and segregation F520, *WT1* (NM_024426.4)

c.[1432+4C>T];[=], p.[?];[=]


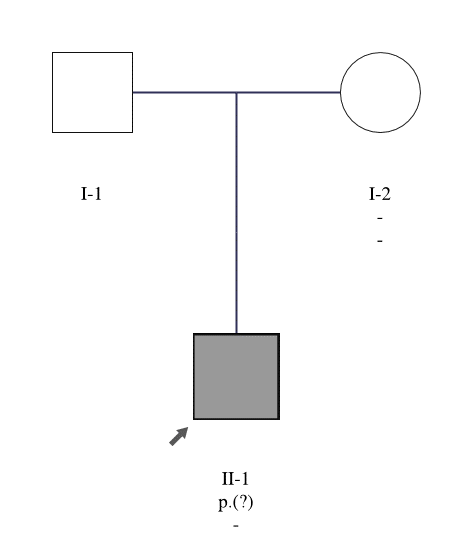


# References

1. Brown EJ, Schlondorff JS, Becker DJ, Tsukaguchi H, Tonna SJ, Uscinski AL *et al*: Mutations in the formin gene INF2 cause focal segmental glomerulosclerosis. *Nat Genet* 2010; **42:** 72-76.
